# Titanium Oxide (TiO_2_)/Polymethylmethacrylate (PMMA) Denture Base Nanocomposites: Mechanical, Viscoelastic and Antibacterial Behavior

**DOI:** 10.3390/ma11071096

**Published:** 2018-06-27

**Authors:** Ali Alrahlah, H. Fouad, Mohamed Hashem, Abdurahman A. Niazy, Abdulhakim AlBadah

**Affiliations:** 1 Restorative Dental Sciences Department, Dentistry College, King Saud University, Riyadh 11545, Saudi Arabia; aalrahlah@ksu.edu.sa; 2 Applied Medical Science Department, Community College, King Saud University, Riyadh 11001, Saudi Arabia; 3 Department of Biomedical Engineering, Faculty of Engineering, Helwan University, Helwan 11792, Egypt; 4 Dental Health Department, College of Applied Medical Sciences, King Saud University, Riyadh 11545, Saudi Arabia; mihashem@ksu.edu.sa; 5 Molecular and Cell Biology (MCB) Laboratory, Prince Naif Health Research Center, College of Dentistry, Riyadh 11437, Saudi Arabia; aaniazy@ksu.edu.sa; 6 Microbiology Research Laboratory, College of Dentistry, King Saud University, Riyadh 11437, Saudi Arabia; Hakim1855@gmail.com

**Keywords:** polymethylmethacrylate, TiO_2_, TGA, creep, nanomechanical, DSC, antibacterial

## Abstract

Currently, polymethylmethacrylate (PMMA) is the most popular denture base material. Most fractures of dentures that occur during function are due to its insufficient mechanical strength. The major drawbacks of PMMA are insufficient ductility, strength, and viscoelastic behavior. The purpose of this study was to evaluate a polymethylmethacrylate denture base material modified with TiO_2_ nanoparticles in terms of nanomechanical, creep-recovery, and relaxation. Additionally, the effects of addition TiO_2_ nanoparticles on the thermal and antimicrobial adhesion behaviors were investigated. Differential scanning calorimetry and thermogravimetric analysis indicated that the effect of small amounts of TiO_2_ nanoparticles (1 wt. %, 2 wt. %, and 3 wt. %) on the degradation behavior of PMMA denture bases was insignificant. The nanomechanical test results of the PMMA and PMMA/TiO_2_ nanocomposites indicated that the hardness and modulus in the nanoscale range improved due to TiO_2_ addition. At a 1200-nm penetration depth, the modulus increased by 10%, 16%, and 29% and hardness increased by 18%, 24%, and 35% with the addition of 1 wt. %, 2 wt. %, and 3 wt. % TiO_2_, respectively. Furthermore, the creep-recovery and relaxation behaviors of PMMA were significantly improved due to the addition of TiO_2_. The creep strain decreased from 1.41% to 1.06%, 0.66%, and 0.49% with the addition of 1 wt. %, 2 wt. %, and 3 wt. % TiO_2_, respectively. The relaxation test results showed that the initial stress under 1% strain improved to 19.9, 21.2, and 22 MPa with the addition of 1 wt. %, 2 wt. %, and 3 wt. % TiO_2_, respectively. The improvement in the nanohardness, modulus, creep recovery, and relaxation behavior of PMMA due to the addition of TiO_2_ nanoparticles indicated the role of the nanoparticles in increasing the PMMA matrix stiffness by reducing its mobility and free volume. TiO_2_ nanoparticles also improved the antimicrobial behavior of PMMA by significantly reducing bacterial adherence with increasing TiO_2_ ratio.

## 1. Introduction

For decades, polymethylmethacrylate (PMMA) has been the most commonly used acrylic resin for denture base fabrication. This resin possesses satisfactory mechanical, physical, and aesthetic characteristics [1]. It also has advantages such as low cost, simple fabrication, low weight, satisfactory aesthetics, good optical properties, biocompatibility, color matching ability, and ease of finishing and polishing [2]. However, PMMA has some limitations such as low surface hardness and poor mechanical resistance against fatigue, impact, and bending. These weaknesses must be considered to improve the performance of PMMA materials as orthodontic appliances and removable partial dentures [3,4,5]. In addition, materials manufactured from PMMA are prone to microbe adhesion, resulting in stomatitis, which influences the palatal mucosa and is commonly recognized as a contagious disease among denture users [6]. Usually, oral hygiene as well as denture cleansing are employed to avoid stomatitis, but for hospitalized and geriatric patients, denture cleansing might be compromised as a result of reduced motor dexterity, cognitive impairment, and memory loss [7]. Previous studies have shown that mechanically employed cleaning methods are inadequate in preventing microorganism adherence on denture bases [8]. 

One major concern facing dentists and patients regarding the use of removable acrylic appliances is the accumulation of plaque due to food retentive configuration and surface porosities, leading to bacterial activity of cariogenic oral flora [9]. Numerous approaches have been proposed to enhance the mechanical properties of acrylic appliances and decrease the risk of dental caries during orthodontic treatment. One recent method is the use of antimicrobial self-cleaning dental composite materials for orthodontic appliances. In this technique, various nanofillers are incorporated into orthodontic materials to enhance their mechanical properties and antimicrobial activity. Several nanoparticles (NPs) such as TiO_2_, SiO_2_, ZnO, CeO_2_, Ag, and halloysite nanotube (HNTs) have been incorporated in numerous biomaterials to induce antimicrobial activity and improve mechanical behavior [10,11,12]. Among these, TiO_2_ has recently gained prominence owing to its noticeable high stability, catalytic effect, availability, white color, efficiency, and low cost [13,14]. Besides, TiO_2_ NPs are non-toxic and chemically inert and have a high refractive index and corrosion resistance as well as high hardness and antibacterial activity under a wide configuration spectrum [15]. Previous studies have shown that even low concentrations of TiO_2_ NPs can induce new physiochemical, optical, and electrical properties, resulting in a favorable new class of nanocomposite materials [15,16,17,18,19]. The effect of TiO_2_ on the physical and mechanical properties of two acrylic resin denture bases has been observed. The results showed that the addition of a very small concentration of TiO_2_ NPs significantly improved the microhardness and impact strength, but adversely affected the flexural strength. Furthermore, TiO_2_ and SiO_2_ NPs have been employed elsewhere as additives to promote the antimicrobial activity of acrylic resins [20]. 

To the best of our knowledge, many studies have investigated the effects of adding of TiO_2_ NPs on the physical, mechanical, and biological behavior of a polymethylmethacrylate matrix. On the other hand, little work has been done on the effects of TiO_2_ NPs on the viscoelastic behavior (creep-recovery and relaxation) behavior of a polymethylmethacrylate matrix. Therefore, the present study is a part of research work that is intended to study the effects of different nanoparticle types and ratios on the properties of a polymethylmethacrylate matrix to give comprehensive data about a polymethylmethacrylate composite. The objectives of the present study were: (1) the fabrication of PMMA/TiO_2_ nanocomposite denture bases; and (2) studying the effects of TiO_2_ NP addition on the nanomechanical, thermal, viscoelastic, and biological characteristics of a PMMA/TiO_2_ nanocomposite. It is expected that these nanocomposites will find widespread commercial application. However, the application of TiO_2_-based nanocomposites in denture base materials is still in its nascent stage, and considerable effort is required to find facile techniques for the synthesis of various nanocomposite structures with effective structure/property relationships.

## 2. Materials and Methods

A commercial heat-curing PMMA denture acrylic was used (Lucitone 550, Dentsply International Inc., PA, USA). The molecular weight of PMMA according to the manufacturer datasheet was 11,000 and its viscosity was 0.20 dL/g. The resin was mixed according to the manufacturer’s instructions and packed into the mold space when the resin mix was in a doughy stage. TiO_2_ NPs were supplied by NaBond Company, China. According to the manufacturer’s datasheet of the material, the TiO_2_ NPs had a surface area of 30 m^2^/g and average sizes of 80–100 nm. To fabricate the PMMA/TiO_2_ specimens, different ratios of TiO_2_ NPs were carefully weighed using a sensitive balance (six digits). Thin discs of PMMA/TiO_2_ nanocomposites were manufactured with TiO_2_ ratios of 1 wt. %, 2 wt. %, and 3 wt. % as the denture base specimens. The specimens were thin and circular with a diameter of 5 cm and a thickness of 1 mm. The discs were cut to the desired shape for each test.

Scanning electron microscopy (SEM; FE-SEM-JEOL GSM-6610LV) equipped with energy-dispersive X-ray spectroscopy was used to characterize the fracture surface morphology of the PMMA/TiO_2_ nanocomposites. The specimens were coated with gold and examined at a voltage of 20 kV.

The structure of the PMMA/TiO_2_ nanocomposites was analyzed by Fourier transform infrared (FTIR, Bruker, TENSOR Series FT-IR Spectrometer, Germany) spectroscopy in the absorbance mode. The spectra were recorded from 500 to 4000 cm^−1^.

The effects of TiO_2_ NP addition on the thermal behavior of PMMA were examined using differential scanning calorimetry (DSC)/thermogravimetric analysis (TGA; Model SDT-Q600, TA-Instrument, USA). The PMMA/TiO_2_ nanocomposite specimens were cut into small parts weighing 7 mg, sealed in an aluminum pan, then heated at a rate of 10 °C/min to 600 °C under nitrogen. The thermal behavior data were obtained by the setup software.

The nanomechanical characteristics of the PMMA/TiO_2_ specimens were measured using a universal nanomechanical tester (Bruker, Campbell, CA, USA). To reduce the vibration during measurements, the machine was equipped with an anti-vibration isolation table. Nanohardness and elastic modulus were measured by a nanoindentation machine at room temperature. Hardness and modulus are functions of maximum applied load and contact penetration depth. During the test, a holding time of 15 s was considered between the loading and unloading processes to reduce the effects of creep.

The creep-recovery and relaxation behaviors of the PMMA/TiO_2_ nanocomposites were characterized by dynamic mechanical analysis using a RSA-G2 analyzer (TA instruments, USA). Creep tests were conducted at different loads (10, 15, and 20 N) and 37 °C (human body temperature) for 240 min each of loading and unloading to determine the influence of loading conditions on the creep behavior of the tested materials. The relaxation behavior of the tested materials was also investigated using the RSA-G2 analyzer. The tests were performed under 1% strain for 180 min and the resulting stress values were recorded. Furthermore, the creep behavior of the tested materials could be defined in terms of creep compliance, j(t), which represents the material strain with time divided by applied stress as the following Equation:

j(t) = ɛ(t)/σ(1)

where ɛ(t) is defined as the recovery strain at any time after load removal and σ is the stress applied on the tested material. The creep compliance at recovery was calculated as a function of recovery time after load removal, according to the following equation:

j(t) = (ɛ_total_(t) − ɛ_remaining_(t))/σ (2)

### Antibacterial Adhesion Activity

The scaffold was composed of PMMA sheets with different TiO_2_ contents (0 wt. %, 1 wt. %, and 3 wt. %). The sheet was cut using a toothed saw into strips of 5 mm × 10 mm. These strips were then soaked in 70% ethanol for 1 h and washed three times in sterile deionized (DI) H_2_O for 15 min. The scaffold strips were stored in a sterile petri dish until use.

In a 24-well plate, each well was filled with 1 mL nutrient broth. Gram-positive and Gram-negative strong biofilm formers and medically important bacteria were chosen: *Enterococcus faecalis* as the Gram-positive species and *Pseudomonas aeruginosa* strain PAO1 as the Gram-negative one. Each well in the plate was inoculated with 1 µL bacterial samples standardized at 0.5 OD A at OD600 nm. The plate was incubated at 37 °C for 48 h. All samples were prepared in triplicate.

Each strip was then removed from the well and dipped into sterile DI water three times to remove any unattached excess bacteria. The strips were transferred into sterile 1.5 mL microcentrifuge tubes containing 1 mL sterile DI water. Each tube was vortexed vigorously for 1 min to remove the attached bacteria. A serial dilution was performed and 1 mL samples were plated onto nutrient agar overnight. The next day, the colony forming units per mL (C.F.U./mL) of the inoculum and plate were estimated, and the average for the three plates in each triplicate set was reported.

## 3. Results and Discussions

### 3.1. Fracture Surface Morphology

**Figure 1:**
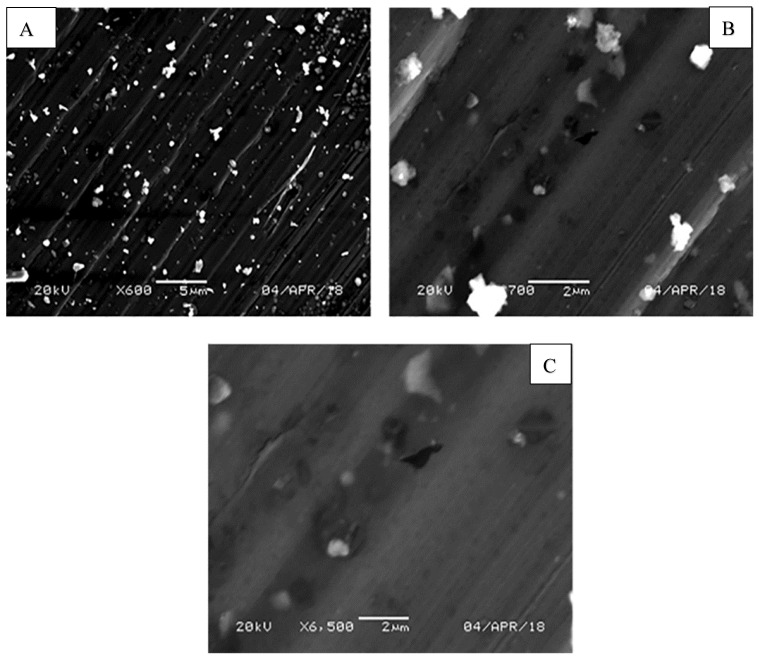
Morphology of PMMA/3 wt. % TiO_2_ nanocomposites at different magnifications (**A**) X600, (**B**) X2700 and (**C**) X6500.

The surface morphology of the PMMA/TiO_2_ nanocomposite specimen containing 3 wt. % TiO_2_ at different magnifications is shown in Figure 1. The TiO_2_ NPs were well distributed in the nanocomposite matrix. Although the NPs were in the nanoscale range according to the manufacturer (80–100 nanometers), the SEM images showed that some TiO_2_ NPs underwent some agglomeration, but were still in the nanoscale with a maximum size of 500 nm. This agglomeration was attributed to the tendency of TiO_2_ NPs to decrease their contact surface with PMMA. The TiO_2_ NPs appeared as bright points in the PMMA matrix. This good distribution of TiO_2_ NPs helped improve the nanocomposite behavior. 

### 3.2. Fourier Transform Infrared Spectroscopy

**Figure 2:**
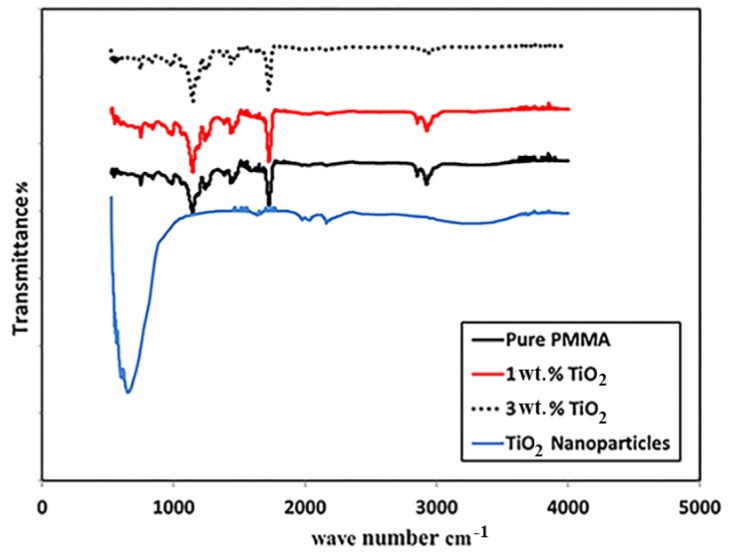
Fourier transform infrared spectra of the pure PMMA and PMMA/2 wt. % TiO_2_ and the PMMA/3 wt. % TiO_2_ nanocomposites.

The FTIR spectra of the TiO_2_ NPs, neat PMMA, and its nanocomposites are shown in Figure 2. For pure TiO_2_, the characteristic band of M–O was observed around 600 cm^−1^, with a band around 2100 cm^−1^ corresponding to C–O, which was attributed to ambient contamination. For neat PMMA, absorption bands were observed at 2993, 1730, and 1420 cm^−1^, which are characteristic bands for PMMA [16]. The band at 3100 cm^−1^ could be assigned to the stretching vibration of Ti–OH and O–H formed by the C=O of PMMA. The addition of TiO_2_ in PMMA did not change the functional characteristics of the composite as no new absorption bands were observed [16]. This was because the PMMA and TiO_2_ were physically mixed; thus, no changes occurred in the chemical structures of the composites. Additionally, the unclear beak of TiO_2_ in the PMMA resin may be due to the low concentration of the nanoparticles.

### 3.3. Thermal Behavior

**Figure 3:**
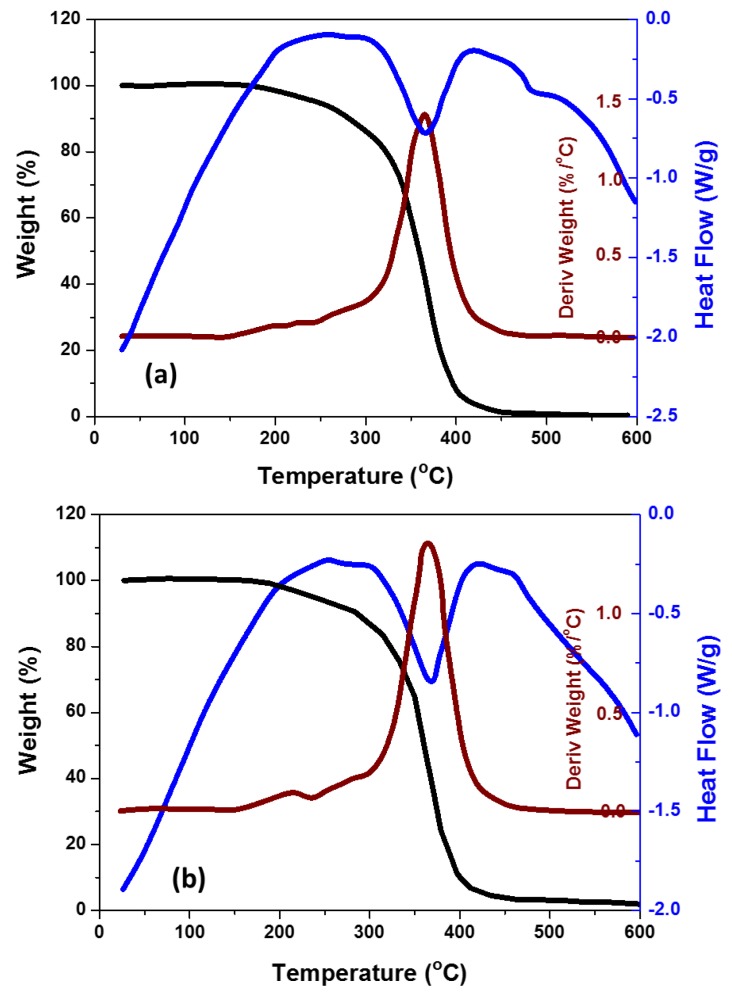
Differential scanning calorimetry and thermogravimetric analysis of (**a**) pure PMMA and (**b**) PMMA/2 wt. % TiO_2_.

The effects of the addition of TiO_2_ NPs (0 wt. % and 2 wt. %) on the thermal behavior of PMMA denture bases investigated at a heating rate of 10 °C/min are shown in Figure 3. The results are represented in the form of glass transition, degradation temperatures, and rate. The glass transition temperature, thermal and degradation temperature of the nanocomposites were shown to slightly increase with an increase in the weight percentage of TiO_2_ in the composite. The glass transition temperature of PMMA increased from 365 °C to 368 °C due to the addition of 2 wt. % TiO_2_. Additionally, the results showed that the degradation of PMMA + 2 wt. % TiO_2_ started at 170 °C, compared to 165 °C for pure PMMA. This can be attributed to the incorporated water evaporation and decomposition of the peroxo groups. It was also observed from Figure 3 that the PMMA and its nanocomposites were completely degraded at 400 °C. This can be attributed to the rupture of the main chain and to the terminal vinyl group decomposition. Slight differences were observed between the degradation behaviors of the PMMA/TiO_2_ nanocomposites and pure PMMA. This slight effect on adding TiO_2_ NPs in the PMMA matrix could be due to the low content of NPs in the matrix and to the small size and weight of the DSC specimens. In other words, the addition of TiO_2_ nanofillers did not have an influence on the mobility of the PMMA chain segments, leading to nearly the same values of glass transition and degradation temperatures for the tested nanocomposites. Similar results have been reported previously [21,22], wherein the DSC and TGA results showed negligible changes with the addition of small concentrations of TiO_2_. 

### 3.4. Nanomechanical Behavior

**Figure 4:**
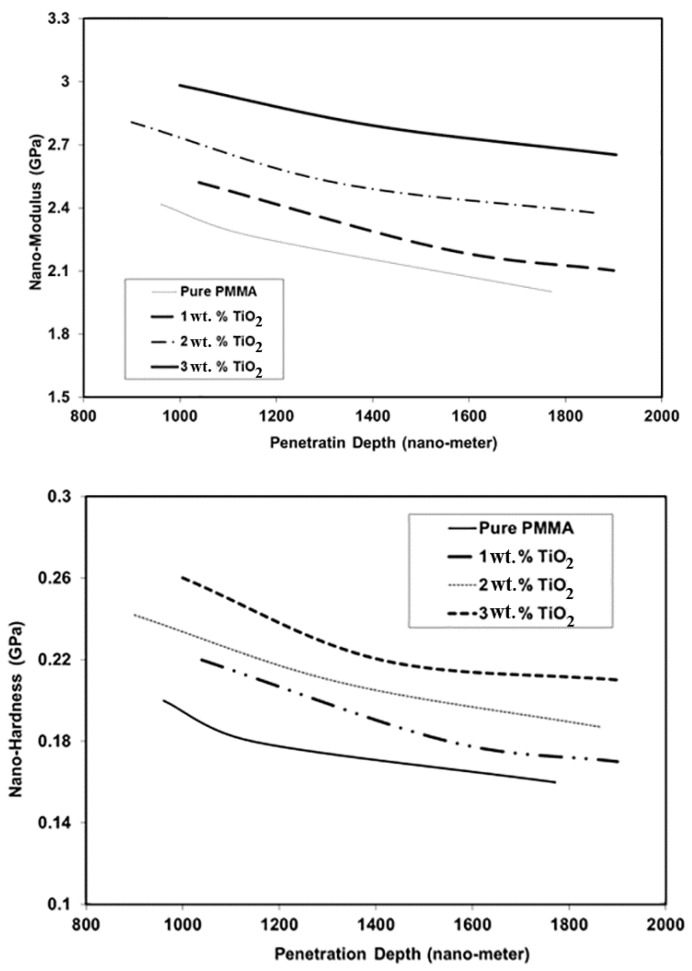
Nanomechanical properties of PMMA and its nanocomposites at different penetration depths for 0wt, 1 wt. %, 2 wt. %, and 3 wt. % TiO_2_ NPs.

Figure 4 shows the effects of the addition of TiO_2_ NPs on the nanomechanical behavior of PMMA. Nanomechanical properties such as hardness and modulus of elasticity were measured at the surface of the tested materials. These properties are functions of load, penetration depth, and indenter type and shape. The results showed that both hardness and modulus increased with the addition of the rigid NPs. For example, the measured modulus at a penetration depth of 1200 nm increased by 10%, 16%, and 29% due to the addition of 1 wt. %, 2 wt. %, and 3 wt. % TiO_2_ NPs, respectively. Similar results were obtained for hardness, with the overall values increasing by 18%, 24%, and 35%, respectively, at the same penetration depth. The observed improvement in hardness and modulus with the addition of TiO_2_ NPs could be attributed to the improvement in the stiffness of the PMMA matrix due to the reduction in its free volume and molecular mobility [23]. Similar results have been obtained for PMMA and other semi-crystalline polymers in other studies; the mechanical properties of these materials were found to improve with the addition of NPs [23,24]. Figure 4 also shows that the nanomechanical properties of PMMA and its nanocomposites degraded with increasing penetration depth. This indicated that the crosslinking at the surface layer of the material was greater than that in the material’s internal layers, resulting in an improvement in the modulus and hardness at the surface. Similar results were obtained for other polymers, with the nanomechanical properties at the surface showing higher values than those in the core [23,24]. The variation of nanohardness (H in MPa) with penetration depth (in nm) for different TiO_2_ ratios can be represented as the following equation with R^2^ = 0.96.

$$H=2.2x{\left(h\right)}^{-0.35}+0.2\left(\mathrm{wt}.\;\%\right)$$

where wt. % is 0, 1, 2 and 3. 

### 3.5. Creep Recovery

**Figure 5:**
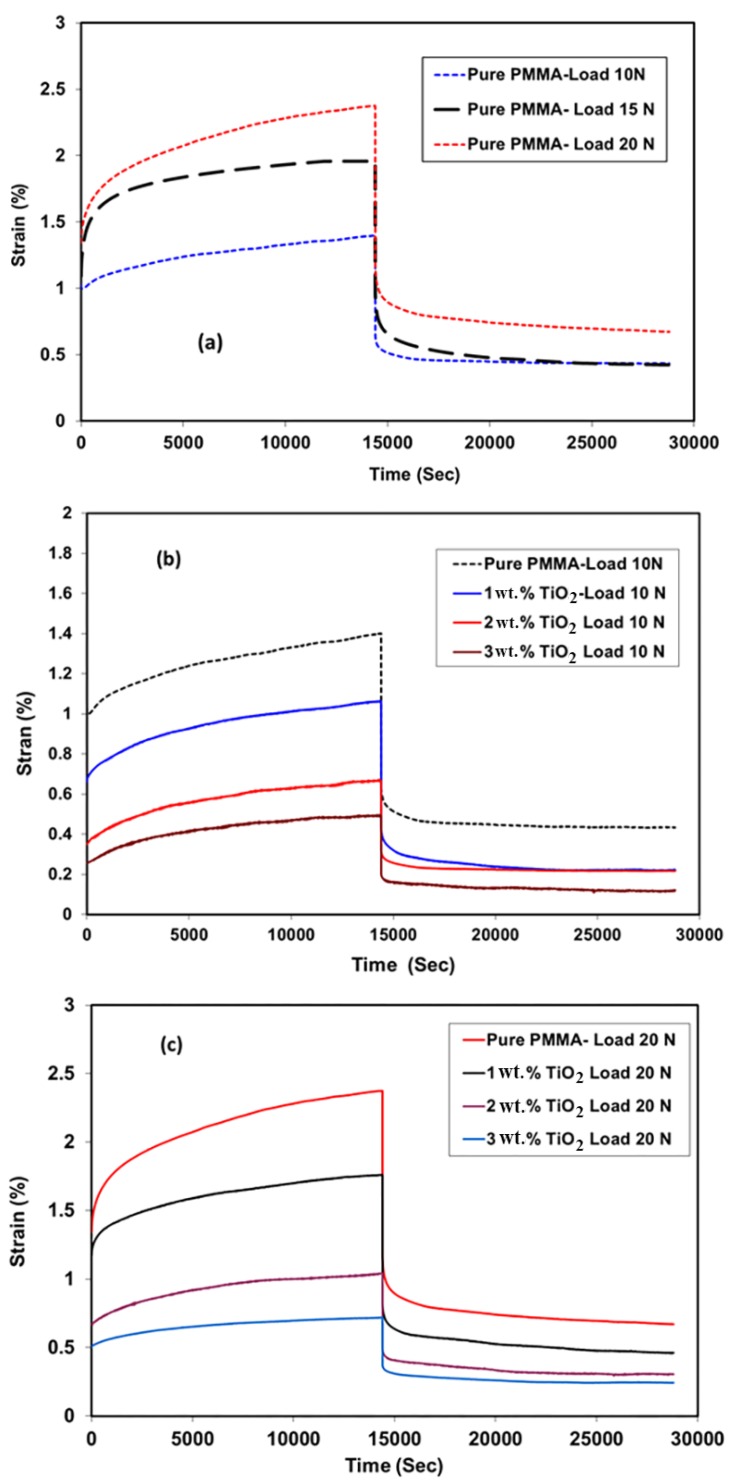
Creep-recovery behavior of PMMA and its nanocomposites at different loads for 0 wt. %, 1 wt. %, 2 wt. %, and 3 wt. % TiO_2_ NPs.

The effects of the addition of TiO_2_ NPs on the creep and recovery behaviors of PMMA is shown in Figure 5. The results are presented as creep and recovery strains against time at different loads for PMMA and its nanocomposites. The creep curves showed that the material initially underwent elastic deformation in a very short time as the strain was slowly increased at constant stress. Following the removal of load, part of the elastic strain rapidly recovered, while the other creep strain portion slowly recovered with time; the remaining strain was considered as the permanent deformation. Figure 5a describes the effects of applied load on the creep behavior of pure PMMA. The creep strain increased as a result of the increasing applied load [25]. The creep strain after 240 min increased from 1.4% to 2% and 2.4% when the applied load was increased from 10 to 15 and 20 N, respectively. Figure 5b,c show the effects of adding TiO_2_ NPs on the creep strain of PMMA at applied loads of 10 and 20 N, respectively. The results showed that the creep strain decreased from 1.4% to 1.1%, 0.7%, and 0.5% due to the addition of 1 wt. %, 2 wt. %, and 3 wt. % TiO_2_, respectively, at a load of 10 N. This improvement in the creep behavior of the PMMA denture bases indicated a longer working life for the dentures. 

**Figure 6:**
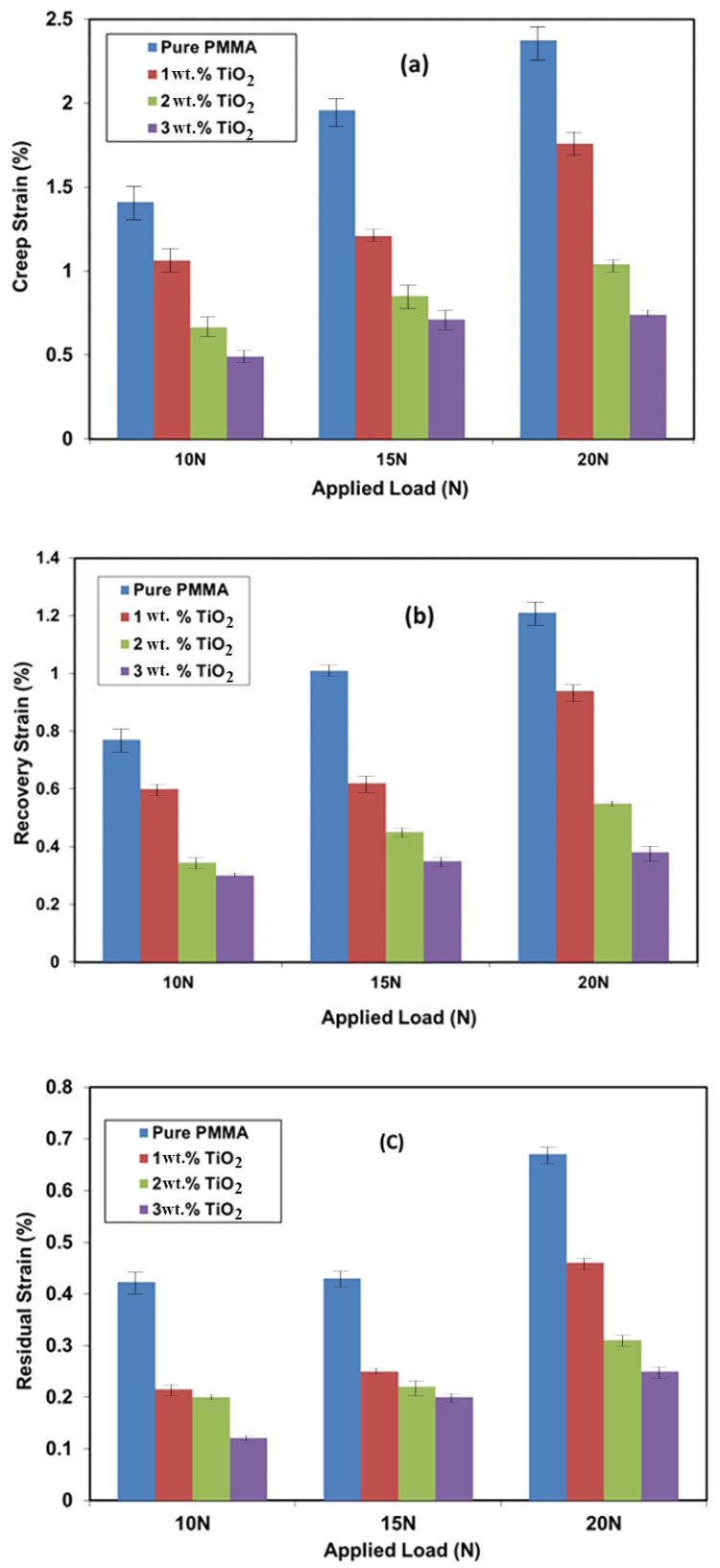
(**a**) Creep, (**b**) recovery and (**c**) residual strain of PMMA and its nanocomposites at different loads.

In addition, the strain values decreased from 2.4% to 1.8%, 1.1%, and 0.8%, respectively, when the applied load was increased to 20 N. The reduction in creep strain due to the presence of TiO_2_ NPs could be because these NPs increase the stiffness of the PMMA matrix, thus reducing its free volume and molecular mobility. Similar results were also obtained for PMMA and other semi-crystalline polymers where the creep behavior improved with the addition of different NPs [26]. The effects of adding TiO_2_ to PMMA on recovery strain (just after removing the load) and residual strain after removing the load within 240 min showed the same trend as that for the creep strain. Figure 6 summarizes the effects of load and TiO_2_ NP addition on the creep, recovery, and residual strain of the materials under investigation.

### 3.6. Relaxation Test

**Figure 7:**
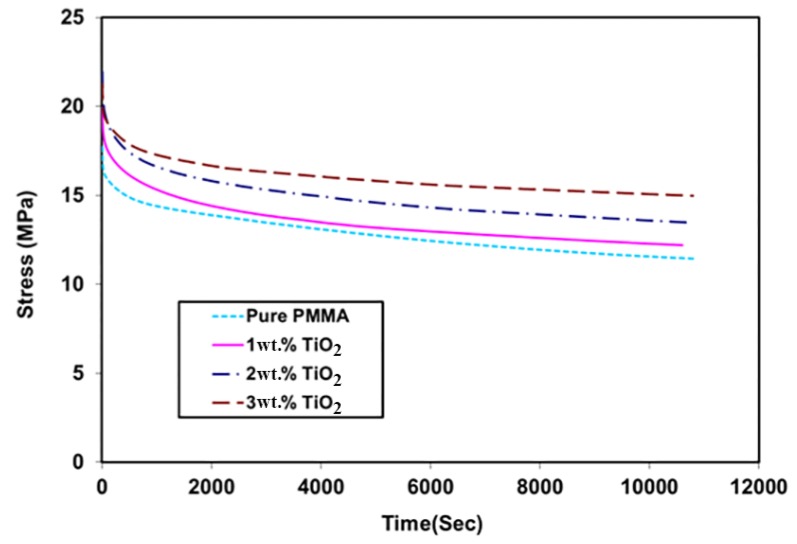
Relaxation behavior of PMMA and its nanocomposites (0 wt. %, 1 wt. %, 2 wt. %, and 3 wt. % TiO_2_ NPs) at 1% strain.

Figure 7 shows the effects of TiO_2_ NP addition on the relaxation behavior of PMMA as a function of time under 1% total strain. The results indicated that all materials under consideration responded to the strain with stress values of up to 17.7 MPa for pure PMMA. This resulting stress decreased with time; after 3 h, the resulting stress value was about 30% of its initial value. Due to the addition of TiO_2_, the resulting stress on the PMMA nanocomposite became higher than that on pure PMMA. This could be considered as an improvement in the nanocomposite properties, as indicated by the nanomechanical and creep behavior. As mentioned earlier, the presence of rigid TiO_2_ NPs increased the stiffness of the PMMA matrix and reduced its free volume and molecular mobility [27,28]. The values of initial stress under 1% strain were 19.9, 21.2, and 22 MPa with the addition of 1 wt. %, 2 wt. %, and 3 wt. % TiO_2_, respectively. For the PMMA/3 wt. % TiO_2_ nanocomposite, the stress value at the end of the test was 17.5 MPa (decreased by 20%). 

### 3.7. Creep Compliance

**Figure 8:**
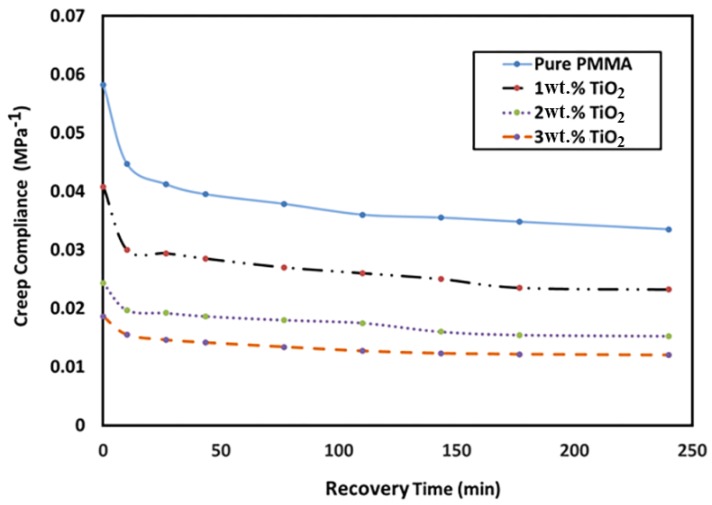
Variation in creep compliance with recovery time and TiO_2_ NP loading ratio (0 wt. % 1 wt. %, 2 wt. % and 3 wt. % TiO_2_ NPs).

Figure 8 shows the variation in recovery creep compliance with respect to the TiO_2_ NP loading ratio and recovery time. The results indicated that the creep compliance values decreased with recovery time for PMMA and its nanocomposites. Furthermore, the creep compliance decreased (improved) significantly as the TiO_2_ NP content increased. This could be attributed to the improvement in the PMMA stiffness due to the addition of nanoparticles. For example, the value of creep compliance decreased from 0.06 to 0.019 with the addition of 3 wt. % TiO_2_.

## 4. Antibacterial Adhesion

**Figure 9:**
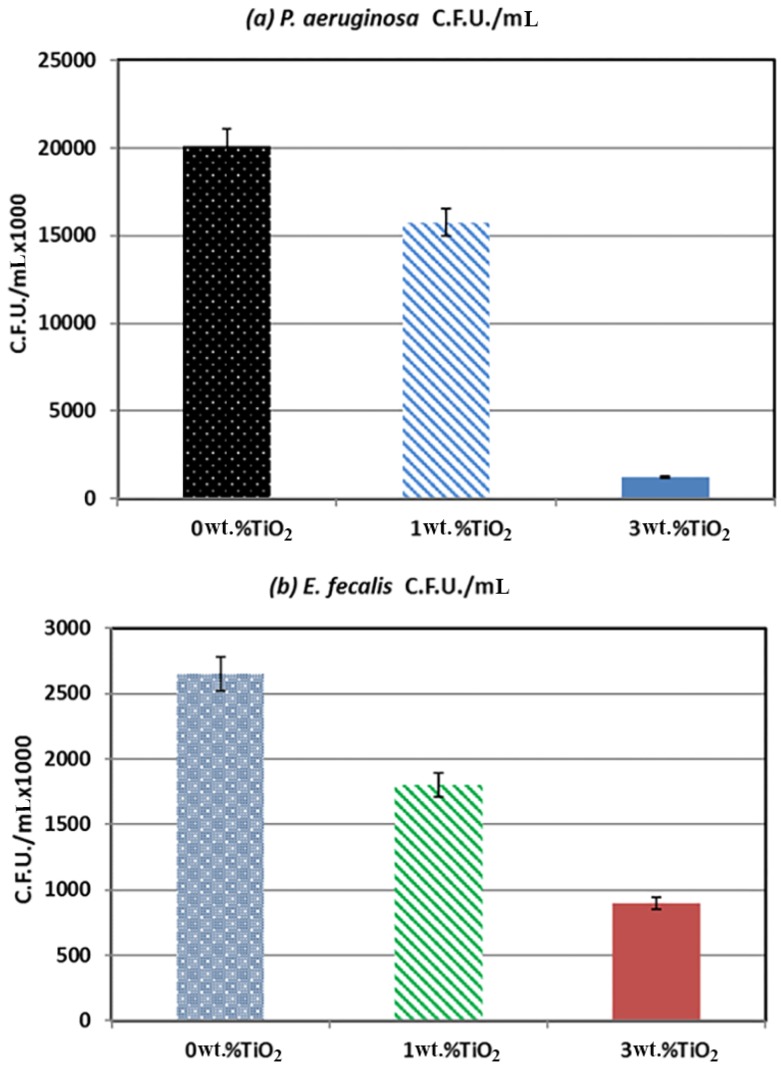
(**a**) Attachment of *P. aeruginosa* cells in colony forming units per mL (C.F.U./mL) to PMMA composites containing 0 wt. %, 1 wt. % and 3 wt. % TiO_2_. (**b**) Attachment of *E. faecalis* cells in C.F.U./mL to PMMA composites containing 0 wt. %, 1 wt. %, and 3 wt. % TiO_2_.

PMMA scaffolds with TiO_2_ concentrations of 0 wt. % (control), 1 wt. % (minimum), and 3 wt. % (maximum) were used for the microbial adhesion analysis. As shown in Figure 9, *E. faecalis* showed a much smaller drop in attached cells than *P. aeruginosa* with the PMMA scaffold. *E. faecalis* with 0%, 1%, and 3% TiO_2_ contained 2.65 × 10^6^, 1.8 × 10^6^, and 9 × 10^5^ C.F.U./mL, respectively. The scaffold strip showed an attached-cell drop rate of about 38% when the TiO_2_ content was increased from 0% to 1%, and when the TiO_2_ content was increased from 1% to 3%, there was a 50% drop in the attached cells.

**Figure 10:**
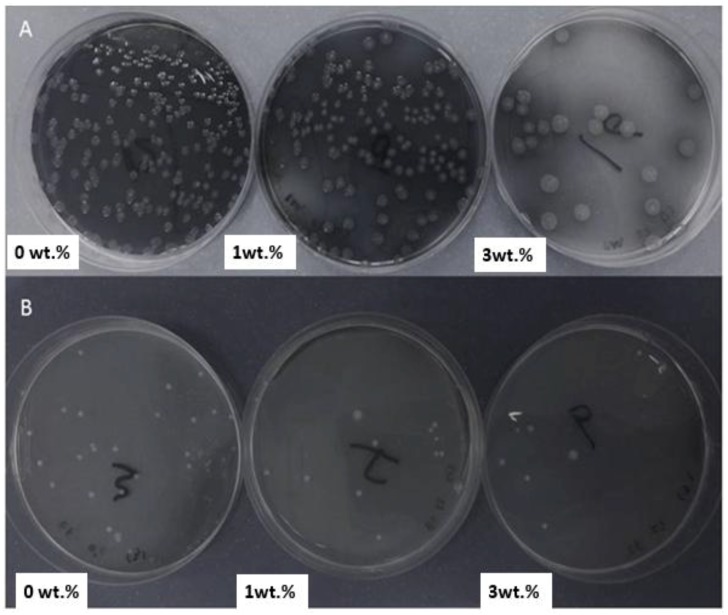
(**A**) C.F.U. of *P. aeruginosa* on nutrient agar after growing it on PMMA composites containing 0 wt. %, 1 wt. %, and 3 wt. % TiO_2_ for 48 h. (**B**) C.F.U. of *E. faecalis* after growing it on PMMA composites containing 0 wt. %, 1 wt. %, and 3 wt. % TiO_2_.

With *P. aeruginosa*, PMMA with 0 wt. % TiO_2_ contained 2.01 × 10^7^ attached cells, while the scaffold strip containing 1 wt. % TiO_2_ had 1.57 × 10^7^ attached cells, showing a 12% drop. The scaffold strip containing 3 wt. % TiO_2_ had 1.25 × 10^6^ attached cells, indicating a sharp drop of about 92% (Figure 10). Although the use of high concentrations of TiO_2_ leads to toxicity, small amounts have been proven to be very helpful for implants [29]. It has been previously reported that the addition of TiO_2_ to PMMA composites improved the quality of the material by reducing bacterial attachment to its surface, thereby reducing the probability of bacterial biofilm formation, which is the leading cause of bacterial infection [30,31,32,33]. This statement is also in agreement with other published reports on the anti-adherence properties of TiO_2_, which confirms its medical utility [34,35]. Future studies may be conducted on the cytotoxicity effects of these composites to confirm their biocompatibility. This behavior can be attributed to the fact that TiO_2_ NPs have a photocatalytic biocidal/antiproliferative ability that can be observed by sunlight and fluorescent light as the UV excitation source. This photocatalytic activity produces highly reactive radicals with the ability to react with most of the neighboring organic bodies. Therefore, cTiO_2_ NPs have a widespread usage in the removal of toxic, harmful, or hazardous pollutants and is used as an antibacterial in dentistry [36,37,38].

The key mechanism of the toxicity of nanoparticles (NPs) is said to be via oxidative stress (OS) that damages lipids, carbohydrates, proteins, and DNA [39]. The most threatening is lipid peroxidation, which causes alterations in cell membrane properties which in turn causes disorder vital cellular functions [40]. Xia et al. [41] revealed that the toxicity of NPs might be anticipated from their ability to generate ROS in vitro. Thus, it is evident from the literature that TiO_2_ NPs lead to an increase in the production of reactive oxygen species (ROS) and oxidative products (i.e., lipid peroxidation) as well as the reduction of cellular antioxidants [42,43].

## 5. Conclusions

This study investigated the effect of rigid TiO_2_ NP addition on the thermal (degradation temperature), mechanical (creep recovery, nanohardness, nanomodulus, and relaxation), and biological (antifungal and antimicrobial) behavior of PMMA denture bases. The DSC and TGA results indicated that adding small amounts of TiO_2_ NPs had a slight effect on the thermal behavior of the PMMA denture bases. Results of the nanomechanical test of PMMA and its nanocomposites indicated that the hardness and modulus in the nanoscale range improved with the addition of TiO_2_ rigid NPs. The TiO_2_ works as hard particles that improve the stiffness of the PMMA matrix and modify the surface properties. Furthermore, the creep-recovery and relaxation behaviors of PMMA significantly improved with the addition of TiO_2_ NPs. The observed improvement in the behavior of PMMA with the addition of TiO_2_ NPs indicated that the NPs increased the stiffness of the PMMA matrix owing to the reduction in its molecular mobility and free volume. Finally, the addition of TiO_2_ NPs also improved the antimicrobial behavior of PMMA denture bases by reducing their bacterial adherence ability, confirming the practical significance of the addition of TiO_2_ NPs. The results showed that the addition of TiO2 NPs in small ratios with a good distribution in the PMMA matrix not only improved the mechanical behavior, but also had a wide effect on the removal of toxic or hazardous pollutants and can be used as an antibacterial in dentistry.
